# Three-dimensional ocular surface displacement and strain measurement using fringe projection profilometry and digital image correlation

**DOI:** 10.1117/1.JBO.31.8.086008

**Published:** 2026-08-21

**Authors:** Victoria MacDans, Badrinath Balasubramaniam, Beiwen Li

**Affiliations:** a University of Georgia, School of Environmental, Civil, Agricultural and Mechanical Engineering, Athens, Georgia, United States; b University of Georgia, School of Electrical and Computer Engineering, Athens, Georgia, United States

**Keywords:** fringe projection profilometry, digital image correlation, intraocular pressure, 3D displacement, strain measurement, hydrostatic pressurization

## Abstract

**Significance:**

Accurate measurement of ocular surface deformation under intraocular pressure (IOP) is essential for understanding eye biomechanics and the progression of pressure-related diseases such as glaucoma. However, existing imaging techniques often involve trade-offs among spatial resolution, system complexity, and the ability to capture full-field three-dimensional (3D) deformation on highly curved, compliant tissues.

**Aim:**

We investigate the feasibility of a noncontact measurement framework that combines fringe projection profilometry (FPP) and two-dimensional digital image correlation (2D DIC) to recover full-field 3D displacement and strain on the ocular surface during pressurization.

**Approach:**

An *ex vivo* porcine eye was subjected to controlled hydrostatic pressurization to simulate IOP variation. FPP was used to reconstruct dense 3D surface geometry across successive loading states, whereas 2D DIC was applied to corresponding texture images to estimate in-plane displacements. These measurements were fused through pixel-wise mapping to recover full-field 3D displacement vectors, from which Green–Lagrange strain components were computed and expressed in a spherical coordinate framework.

**Results:**

The proposed framework was able to capture the evolving deformation of the ocular surface, revealing spatially heterogeneous displacement and strain patterns. Localized regions of elevated strain were consistently observed, whereas the deformation fields exhibited smooth temporal progression across loading stages. The recovered strain distributions aligned with expected biomechanical behavior of the ocular structure, suggesting the capability of the method to resolve meaningful deformation features on a curved, compliant surface.

**Conclusions:**

The combined FPP–DIC framework indicates potential as a viable approach for measuring dense 3D displacement and strain on the ocular surface. This feasibility study establishes the potential of structured-light-based methods for ocular biomechanics and lays the foundation for future quantitative validation and *in vivo* applications.

## 1 Introduction

Intraocular pressure (IOP) is the internal pressure that maintains the structural stability of the eye. When IOP deviates from its normal range, it alters the mechanical forces within the eye, leading to deformation of the ocular surface. Such deformations are closely associated with vision-threatening diseases such as glaucoma. For this reason, studying ocular surface deformation under pressure variations is essential for understanding ocular disease development and guiding efforts toward its treatment.^1^^,^^2^ Accurately measuring these deformations, however, remains challenging. The eye is a soft, highly curved biological structure that undergoes subtle changes under small pressure variations. As a result, the induced surface displacements are often extremely small, making it difficult to capture with high fidelity.^2^

The current methods for capturing these deformations primarily involve the use of optical imaging methods due to their noncontact nature. Techniques such as optical coherence tomography (OCT), ultrasound biomicroscopy, and laser scanning are among some of the most widely used methods.^3^^–^^5^ Although useful in this regard, each modality presents its own trade-offs. Optical coherence tomography (OCT), for example, provides high-resolution cross-sectional imaging. However, because it captures images in a depth-resolved, sequential manner, its ability to recover consistent full-field deformation is limited, particularly under dynamic conditions. Ultrasound biomicroscopy, on the other hand, enables imaging of deeper tissue structures and can provide more stable structural information; however, it is constrained by lower spatial resolution. Similarly, laser scanning, although capable of generating detailed surface geometry, is often limited by acquisition speed, sensitivity to motion, and measurement consistency.^6^^,^^7^ As a result, a gap remains in identifying a noncontact imaging technique that can simultaneously capture dense three-dimensional (3D) surface geometry and full-field deformation without compromising key performance factors such as spatial resolution, imaging speed, and system complexity.

Recent studies point to the combination of fringe projection profilometry (FPP) with two-dimensional digital image correlation (2D DIC) as a promising solution for achieving this.^8^ FPP is a structured-light technique capable of reconstructing high-resolution and densely sampled 3D surface geometry. In parallel, 2D DIC provides robust, subpixel tracking of surface motion from grayscale images. By integrating these two measurements, it becomes possible to recover full-field 3D displacement through mapping image-based motion onto reconstructed geometry.^9^ Although this combined approach has demonstrated effectiveness in other applications, such as structural beam deformation and deformation analysis of molded pulp materials,^10^^,^^11^ its suitability for measuring deformation of the ocular surface remains largely unexplored. This study therefore investigates the use of an FPP–DIC framework for measuring ocular surface deformation under controlled pressurization conditions. The aim is to assess whether the proposed approach can capture meaningful displacement and strain patterns consistent with established understanding of ocular biomechanics. This, in turn, allows evaluation of its potential as a noncontact measurement tool for ocular surface analysis.

The remainder of this paper is organized as follows. Section 2 presents the theoretical framework of the proposed FPP–DIC approach. Section 3 describes the methods and experimental setup used to simulate intraocular pressurization of the porcine eye. Section 4 presents the reconstructed surfaces, displacement fields, and strain measurements obtained during the experiment. Section 5 discusses the implications and limitations of the proposed method.

## 2 Theory

### 2.1 Theoretical Principles of Fringe Projection Profilometry

FPP is a structured-light optical technique that works by projecting sinusoidal fringe patterns, alternating bright and dark stripes, onto an object’s surface. As these patterns interact with the surface, they deform in response to local height variations. The resulting deformed patterns are analyzed to infer the 3D geometry of the object.^12^^,^^13^ A typical FPP system consists of two primary components: a digital projector and a high-resolution camera positioned at fixed angles relative to one another. Together, these devices form a triangulation geometry with the object being scanned.^14^ During image acquisition, the projector emits phase-shifted defocused binary fringe patterns onto the object. Specifically, a three-step phase-shifting technique is employed. This approach involves projecting three defocused binary fringe patterns with relative phase offsets of 2π/3 sequentially onto the object. The three-step approach was selected to reduce the number of projected fringe patterns and shorten the acquisition time during the dynamic ocular deformation measurements. At the same time, defocusing the binary patterns enabled them to approximate sinusoidal illumination and support accurate phase retrieval without requiring a separate projector gamma-calibration procedure.^15^ Although projection is ongoing, the camera captures the resulting deformed fringe patterns as spatial intensity variations across the image. The spatial intensity distributions corresponding to these three phase steps are expressed as:

$$I_{1}(x,y)=I^{\prime }(x,y)+I^{\prime \prime }(x,y)\cos[\phi (x,y)-\frac{2\pi }{3}],$$ (1)

$$I_{2}(x,y)=I^{\prime }(x,y)+I^{\prime \prime }(x,y)\cos[\phi (x,y)],$$ (2)

$$I_{3}(x,y)=I^{\prime }(x,y)+I^{\prime \prime }(x,y)\cos[\phi (x,y)+\frac{2\pi }{3}],$$ (3)

where $I_{n}(x,y)$ represents the spatial intensity at pixel location (x,y), $I^{\prime }(x,y)$ represents the background intensity component, $I^{\prime \prime }(x,y)$ denotes the fringe modulation amplitude, and ϕ(x,y) corresponds to the phase distribution related to the surface geometry. The parameter N represents the total number of phase-shift steps, whereas n denotes the index of the projected fringe pattern, where n=1,2,…,N.^16^

Solving the three intensity equations yields the wrapped phase,

$$\phi (x,y)=\tan^{-1}(\frac{\sqrt{3}(I_{1}-I_{3})}{2I_{2}-I_{1}-I_{3}}).$$ (4)

Because the arctangent function is periodic, the recovered phase is confined to the interval [−π,π] and contains 2π discontinuities. To obtain a continuous representation of surface geometry, phase unwrapping is applied, correcting these jumps by adding or subtracting 2π multiples to produce a continuous phase. A dual(two)-frequency temporal phase-unwrapping approach is used for phase unwrapping. In this approach, a low-frequency phase is used to determine the fringe order required to unwrap a high-frequency phase.^17^ After the fringe order has been determined, the unwrapped phase is calculated as follows:

Φ(x,y)=ϕ(x,y)+2πk(x,y), (5)

where Φ(x,y) denotes the absolute phase and k(x,y) represents the fringe order determined during the unwrapping process.^16^

This unwrapped phase establishes a unique correspondence between each camera pixel $\left(u_{c},v_{c}\right)$ and its associated projector pixel $u_{p}$. Hence, this phase-based pairing enables triangulation, where the 3D coordinates $[x,y,z{]}^{T}$ are computed from the intersection of the camera viewing ray and the projector illumination ray.

Accurate triangulation, however, requires knowledge of the intrinsic and extrinsic parameters of both devices. These parameters were obtained through system calibration, in which both the camera and projector were modeled as pinhole imaging systems.^14^ Calibration yields the projection matrices:

$$M_{c}=[\begin{array}{cccc} m_{c11} & m_{c12} & m_{c13} & m_{c14}\\ m_{c21} & m_{c22} & m_{c23} & m_{c24}\\ m_{c31} & m_{c32} & m_{c33} & m_{c34} \end{array}],\;M_{p}=[\begin{array}{cccc} m_{p11} & m_{p12} & m_{p13} & m_{p14}\\ m_{p21} & m_{p22} & m_{p23} & m_{p24}\\ m_{p31} & m_{p32} & m_{p33} & m_{p34} \end{array}].$$ (6)

Here, $M_{c}$ and $M_{p}$ denote the projection matrices of the camera and projector, respectively. Using these matrices, the projection relations are expressed as:

$$u_{c}=\frac{m_{c11}x+m_{c12}y+m_{c13}z+m_{c14}}{m_{c31}x+m_{c32}y+m_{c33}z+m_{c34}},$$ (7)

$$v_{c}=\frac{m_{c21}x+m_{c22}y+m_{c23}z+m_{c24}}{m_{c31}x+m_{c32}y+m_{c33}z+m_{c34}},$$ (8)

$$u_{p}=\frac{m_{p11}x+m_{p12}y+m_{p13}z+m_{p14}}{m_{p31}x+m_{p32}y+m_{p33}z+m_{p34}}.$$ (9)

Rearranging these expressions yields a linear system in (x,y,z), which can be written in matrix form as

$$[\begin{array}{c} x\\ y\\ z \end{array}]={\left[\begin{array}{ccc} m_{c11}-u_{c}m_{c31} & m_{c12}-u_{c}m_{c32} & m_{c13}-u_{c}m_{c33}\\ m_{c21}-v_{c}m_{c31} & m_{c22}-v_{c}m_{c32} & m_{c23}-v_{c}m_{c33}\\ m_{p11}-u_{p}m_{p31} & m_{p12}-u_{p}m_{p32} & m_{p13}-u_{p}m_{p33} \end{array}\right]}^{-1}[\begin{array}{c} u_{c}m_{c34}-m_{c14}\\ v_{c}m_{c34}-m_{c24}\\ u_{p}m_{p34}-m_{p14} \end{array}].$$ (10)

Solving this system for all valid correspondences produces a dense 3D point cloud representation of the scanned object.^14^

### 2.2 Texture Image Generation

In addition to the phase information used for geometric reconstruction, the captured fringe sequence also contains intensity information that can be used to generate a texture image of the observed surface. This texture image represents the projector-illuminated grayscale appearance of the object as observed by the camera and provides spatial intensity features for displacement tracking.^11^

The texture image is computed from the captured phase-shifted fringe sequence by combining the average intensity of the fringe patterns ($I^{\prime }$) with the fringe modulation amplitude ($I^{\prime \prime }$). Accordingly, the texture intensity at pixel location (x,y) can be expressed as

$$T(x,y)=I^{\prime }(x,y)+I^{\prime \prime }(x,y).$$ (11)

$I^{\prime }$ can be calculated as

$$I^{\prime }(x,y)=\frac{1}{3}\overset{3}{\underset{n=1}{\sum }}I_{n}(x,y).$$ (12)

Whereas $I^{\prime \prime }(x,y)$ can be computed as:

$$I^{\prime \prime }(x,y)=\frac{\sqrt{3(I_{1}-I_{3}{)}^{2}+{\left(2I_{2}-I_{1}-I_{3}\right)}^{2}}}{3}.$$ (13)

The resulting texture image provides a stable grayscale image across the ocular surface that is suitable for feature tracking.

### 2.3 2D Digital Image Correlation for In-Plane Displacement Estimation

Two-dimensional digital image correlation (2D DIC) is a well-established optical technique for measuring full-field in-plane deformation. It works by tracking the motion of surface features between images acquired before and after deformation. By comparing corresponding regions in these images, the technique enables the estimation of pixel-wise displacement fields with subpixel accuracy.^18^ Accurate tracking, however, requires sufficient spatial intensity variation within the image. As a result, surfaces are typically characterized by random, high-contrast patterns, with artificial speckle patterns applied when natural texture is insufficient.

To acheive 2D DIC, a reference (undeformed) image of the surface is first subdivided into small subsets centered at selected pixel locations. For each subset, the corresponding location in the deformed image is determined by maximizing a correlation criterion that quantifies the similarity between the two image regions. This correlation-based matching establishes the displacement of each subset between the two states.

Let (x,y) denote the coordinates of a pixel in the reference image. After deformation, the same material point appears at the location (x+u(x,y),y+v(x,y)) in the deformed image. The resulting in-plane displacement vector can therefore be expressed as

$$d(x,y)=[\begin{array}{c} u(x,y)\\ v(x,y) \end{array}],$$ (14)

where u(x,y) and v(x,y) represent the horizontal and vertical displacement components, respectively. This displacement field d(x,y) describes the in-plane motion of surface points in the image domain and serves as the fundamental quantity for subsequent deformation and strain analysis.

### 2.4 Fusion of FPP and 2D DIC for 3D Displacement Recovery

FPP provides the 3D geometry of the observed surface, whereas 2D DIC estimates the 2D displacement of image points within the camera coordinate system. Because both measurements are defined within the same image coordinate frame, the tracked pixel locations obtained from DIC can be mapped onto the reconstructed 3D surface to recover full 3D displacement vectors.^19^

Let (x,y) denote the coordinates of a pixel in the reference texture image. Through the FPP reconstruction process, this pixel corresponds to a 3D surface point,

$$P(x,y)=[\begin{array}{c} X(x,y)\\ Y(x,y)\\ Z(x,y) \end{array}].$$ (15)

After deformation, the same surface location appears at the pixel position (x+u(x,y),y+v(x,y)) in the subsequent texture image. To recover the 3D coordinates of this point, the displaced pixel location is mapped onto the reconstructed geometry of the deformed surface. However, because the updated coordinates (x+u,y+v) generally do not coincide with integer pixel positions in the deformed image, the corresponding 3D coordinates cannot be directly retrieved. Instead, they are evaluated through bilinear interpolation of the reconstructed coordinate fields. The spatial coordinates obtained after deformation are therefore given by

$$P^{\prime }(x,y)=[\begin{array}{c} X^{\prime }(x,y)\\ Y^{\prime }(x,y)\\ Z^{\prime }(x,y) \end{array}].$$ (16)

The 3D displacement vector of the surface point is obtained as the difference between the reconstructed coordinates before and after deformation,

$$D(x,y)=P^{\prime }(x,y)-P(x,y)=[\begin{array}{c} U(x,y)\\ V(x,y)\\ W(x,y) \end{array}],$$ (17)

where U(x,y), V(x,y), and W(x,y) denote the displacement components along the X, Y, and Z directions of the reconstructed 3D coordinate system.

**Fig. 1:**
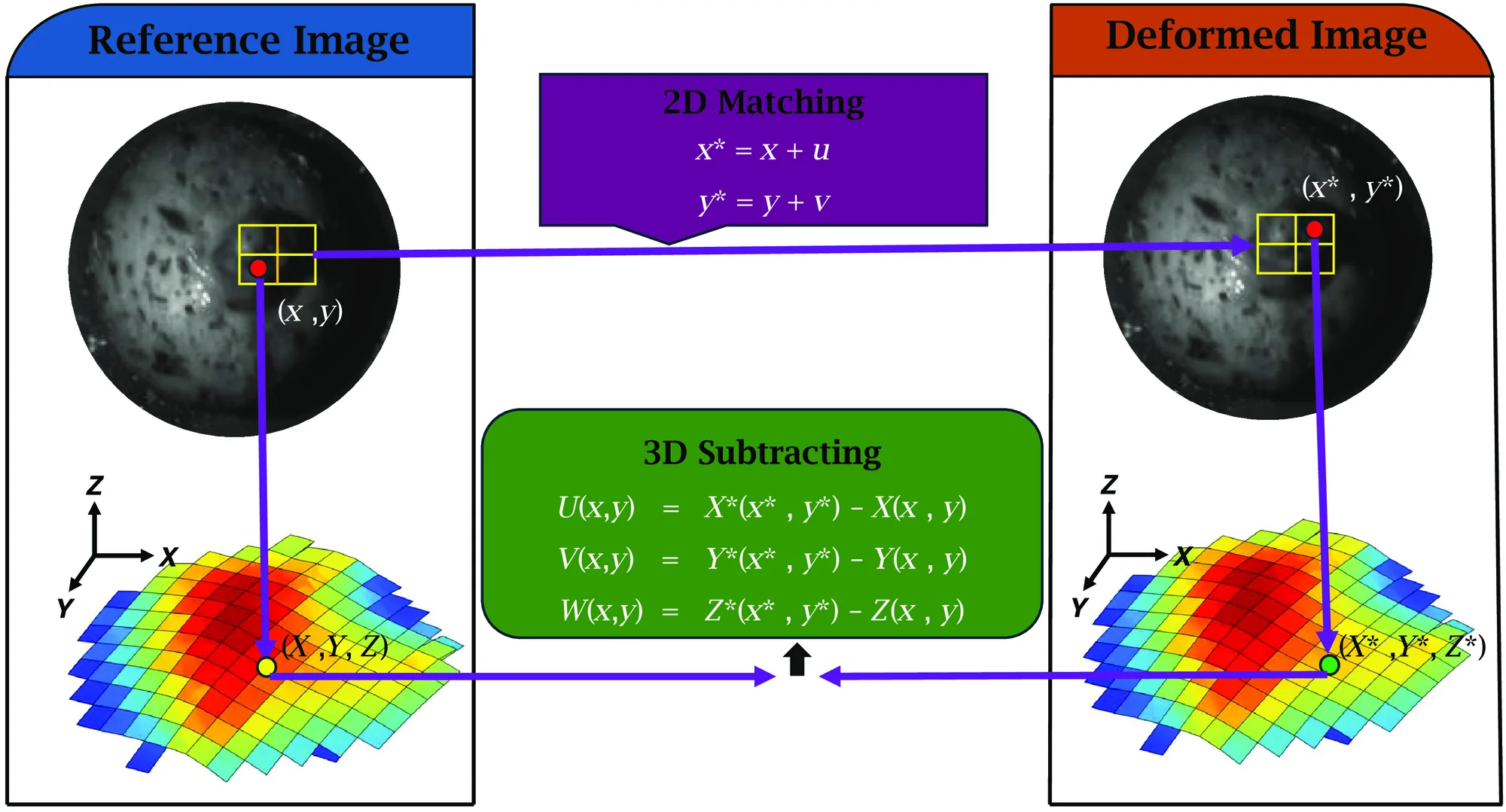
Schematic of the FPP–DIC fusion pipeline for 3D displacement recovery. A surface point at pixel (x,y) in the reference texture image is tracked to $\left(x^{*},y^{*}\right)$ in the deformed image using 2D digital image correlation (DIC). Through FPP reconstruction, each pixel is associated with its corresponding 3D coordinates, yielding (X,Y,Z) in the reference configuration and $\left(X^{*},Y^{*},Z^{*}\right)$ in the deformed configuration (evaluated via bilinear interpolation at subpixel locations). The full-field 3D displacement components (U,V,W) are then obtained by subtracting the reference coordinates from the deformed coordinates.

Applying this mapping procedure to all valid pixels yields a dense full-field three-dimensional displacement field over the ocular surface. This fusion of FPP-based surface reconstruction with image-plane displacement estimation from DIC enables measurement of surface deformation using a single calibrated camera–projector system. A schematic representation of this mapping and subtraction procedure is illustrated in Fig. 1.

### 2.5 Strain Estimation and Coordinate Transformation

Once the 3D displacement field is obtained, surface strain can be calculated from the displacement components. To compute the Green–Lagrange strain, the in-surface strain components are expressed as

$$E_{\mathrm{xx}}=\frac{\partial U}{\partial x}+\frac{1}{2}[{\left(\frac{\partial U}{\partial x}\right)}^{2}+{\left(\frac{\partial V}{\partial x}\right)}^{2}+{\left(\frac{\partial W}{\partial x}\right)}^{2}],$$ (18)

$$E_{\mathrm{yy}}=\frac{\partial V}{\partial y}+\frac{1}{2}[{\left(\frac{\partial U}{\partial y}\right)}^{2}+{\left(\frac{\partial V}{\partial y}\right)}^{2}+{\left(\frac{\partial W}{\partial y}\right)}^{2}],$$ (19)

$$E_{\mathrm{xy}}=E_{\mathrm{yx}}=\frac{1}{2}(\frac{\partial U}{\partial y}+\frac{\partial V}{\partial x})+\frac{1}{2}[\frac{\partial U}{\partial x}\frac{\partial U}{\partial y}+\frac{\partial V}{\partial x}\frac{\partial V}{\partial y}+\frac{\partial W}{\partial x}\frac{\partial W}{\partial y}].$$ (20)

Here, $E_{\mathrm{xx}}$ and $E_{\mathrm{yy}}$ represent the normal strain components, whereas $E_{\mathrm{xy}}$ denotes the in-plane shear strain. The Cartesian strain tensor is therefore expressed as

$$E_{\text{cart}}=[\begin{array}{ccc} E_{\mathrm{xx}} & E_{\mathrm{xy}} & 0\\ E_{\mathrm{xy}} & E_{\mathrm{yy}} & 0\\ 0 & 0 & 0 \end{array}].$$ (21)

To express the strain in spherical coordinates, a coordinate transformation is applied as

$$E_{\mathrm{sph}}=TE_{\text{cart}}T^{T},$$ (22)

where the transformation matrix T is defined as

$$T=[\begin{array}{ccc} -\sin\;\alpha & 0 & \cos\;\alpha \\ -\sin\;\beta \;\cos\;\alpha & \;\cos\;\beta & -\sin\;\beta \;\sin\;\alpha \\ \cos\;\beta \;\cos\;\alpha & \sin\;\beta & \cos\;\beta \;\sin\;\alpha \end{array}].$$ (23)

After transformation, the spherical strain tensor is expressed as

$$E_{\mathrm{sph}}=[\begin{array}{cc} E_{\theta \theta } & E_{\theta \phi }\\ E_{\theta \phi } & E_{\phi \phi } \end{array}],$$ (24)

where $E_{\theta \theta }$, $E_{\phi \phi }$, and $E_{\theta \phi }$ represent the circumferential strain, meridional strain, and in-plane shear strain, respectively.^20^

## 3 Materials and Methods

**Fig. 2:**
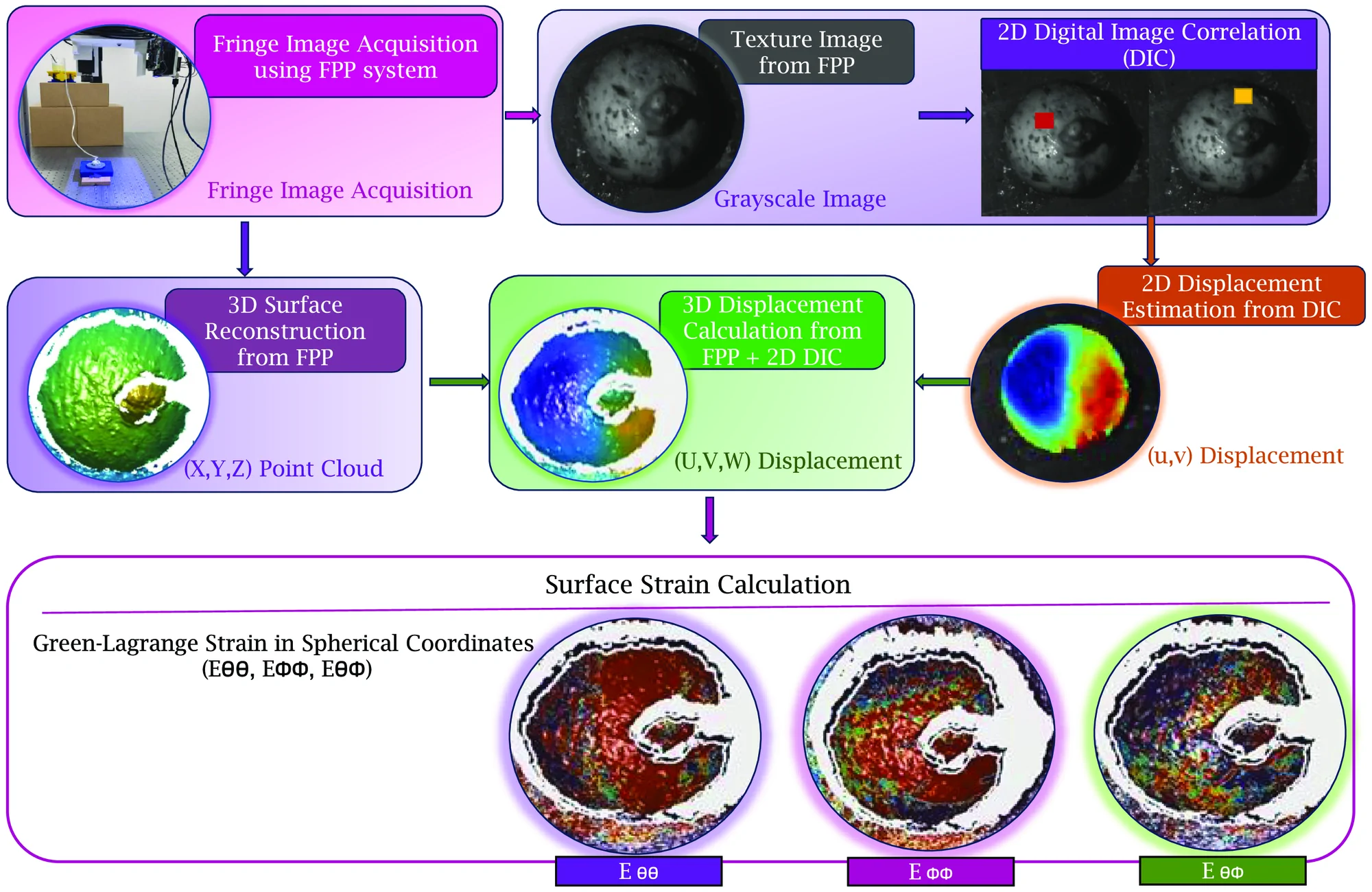
Workflow of the integrated fringe projection profilometry (FPP) and digital image correlation (DIC) framework for measuring 3D ocular surface deformation and strain. In the first stage, defocused binary(sinusoidal) fringe patterns are projected onto the porcine eyeball and captured by a camera to obtain phase-based 3D measurements. The acquired data are processed to generate a reconstructed 3D surface point cloud (X,Y,Z) and corresponding grayscale texture images. The texture images are then analyzed using 2D digital image correlation to estimate in-plane pixel displacements, which are subsequently mapped onto the reconstructed 3D surface to obtain full 3D displacement fields (U,V,W). Finally, the strain tensor components $\left(E_{\theta \theta },E_{\phi \phi },E_{\theta \phi }\right)$ are computed to quantify the mechanical deformation of the ocular surface.

To evaluate the capability of the proposed FPP–DIC framework in capturing ocular surface deformation, this study adopts a multistage measurement pipeline. First, FPP is used to reconstruct the 3D geometry of the ocular surface during controlled pressurization. Next, two-dimensional DIC is applied to corresponding 2D grayscale texture images, obtained from FPP, to track surface displacement over time. Finally, these image-based displacements are mapped onto the reconstructed geometry to recover full-field 3D displacement, from which strain distributions are computed. The overall measurement framework is summarized in Fig. 2

The sections that follows describe the experimental setup and analysis of the system.

### 3.1 Experimental Setup and Analysis

#### 3.1.1 Ocular specimen preparation and intraocular pressure simulation

To evaluate the proposed FPP–DIC framework under controlled mechanical loading, experiments were conducted on an *ex vivo* porcine eyeball. Prior to testing the framework, high-contrast speckle patterns were applied to the eyeball surface to enable effective DIC (Sec. 2.3). Because the proposed framework combines FPP with 2D DIC, the applied speckle pattern was selected to satisfy the requirements of both techniques. Specifically, the pattern provided sufficient random intensity variation for reliable DIC feature tracking while preserving adequate visibility and contrast of the projected fringe patterns required for FPP phase retrieval. Excessive surface patterning could reduce fringe modulation and affect phase recovery, whereas insufficient texture could reduce the reliability of the DIC displacement calculations. The speckle pattern was therefore applied to provide a balance between fringe quality for 3D reconstruction and texture quality for displacement tracking.

**Fig. 3:**
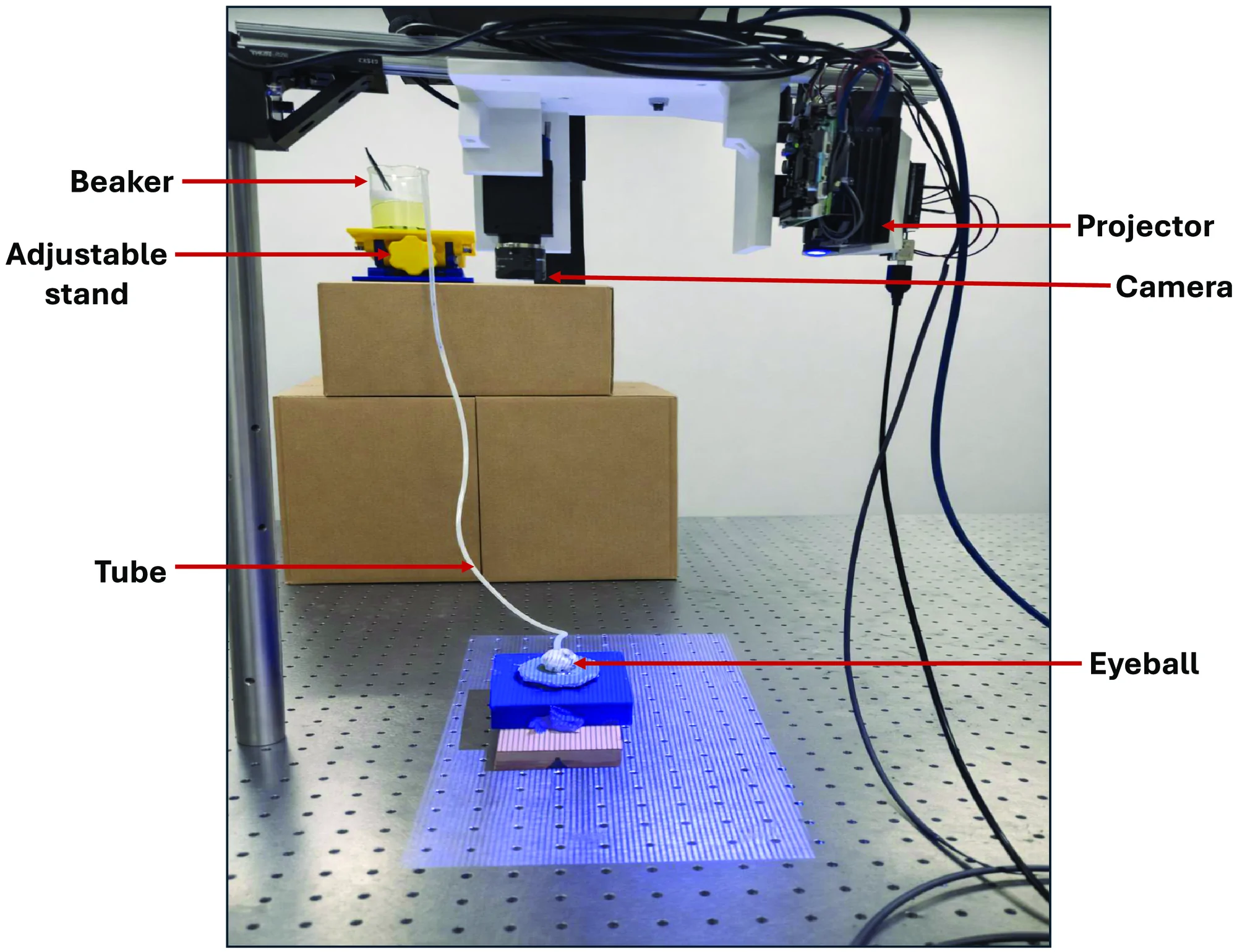
Experimental imaging setup for ocular deformation measurement. The system consists of a calibrated projector–camera pair arranged to form a triangulation geometry with the ocular surface for FPP. A porcine eyeball is mounted on a support platform and connected via tubing to a fluid reservoir. Controlled intraocular pressure is induced by adjusting the height of the reservoir relative to the eyeball.

Following speckle application, intraocular pressure (IOP) was experimentally simulated in the eyeball to induce controlled mechanical deformation. To perform this, the eyeball was first cannulated and securely mounted onto a fixed support to minimize rigid body motion during loading. It was then connected to a hydrostatic pressurization system. This system, consisting of a fluid reservoir, was connected directly to the ocular cavity through a flexible tube, forming a closed hydraulic pathway. The reservoir, a beaker filled with water, was then mounted on an adjustable vertical stand to enable precise control of its elevation relative to the center of the eyeball (Fig. 3). By establishing a controlled height difference between the reservoir and the eyeball, hydrostatic pressure gradient can be generated. As this height is increased, a pressure imbalance is created, driving fluid from the reservoir into the ocular cavity until equilibrium is achieved.^21^ This, in turn, elevates the intraocular pressure within the eye, causing it to expand, resulting in measurable surface displacement across the ocular surface.

#### 3.1.2 FPP system geometric validation

To capture the deformation of the ocular surface under pressurization, image acquisition was performed using the FPP system described in Sec. 2.1. The system comprised of a Texas Instruments LightCrafter 4500 projector and a FLIR camera (model: Grasshopper3 GS3-U3-23S6M), arranged in a triangulated configuration with respect to the eyeball, as illustrated in Fig. 3.

However, before imaging the ocular specimen, the geometric reconstruction accuracy of the FPP system was evaluated using a reference spherical target with a known diameter of 100 mm. This validation was performed to quantify the accuracy of the reconstructed 3D geometry under the same calibrated camera–projector configuration later used for the ocular measurements. To perform the validation, the spherical target was first positioned within the common measurement area of the camera and projector, where it was illuminated by the projected fringe patterns and simultaneously observed by the camera. The resulting fringe images were captured and processed using the same phase retrieval, phase-unwrapping, calibration, and 3D reconstruction procedures described in Sec. 2.1.

The reconstructed point cloud of the known spherical target was subsequently compared with an ideal spherical surface using a RANSAC-based sphere-fitting procedure.^22^ The fitted sphere was modeled as

$$(x-x_{0}{)}^{2}+(y-y_{0}{)}^{2}+(z-z_{0}{)}^{2}=r^{2},$$ (25)

where $\left(x_{0},y_{0},z_{0}\right)$ denotes the fitted sphere center and r denotes the fitted sphere radius. After the inlier points were identified, the final sphere parameters were estimated from those points.

The geometric reconstruction accuracy was then evaluated from the radial error between each reconstructed point and the fitted spherical surface. The equation for calculating the radial error is expressed as:

$$E_{\text{radial},i}=\left|\sqrt{(x_{i}-x_{0}{)}^{2}+(y_{i}-y_{0}{)}^{2}+(z_{i}-z_{0}{)}^{2}}-r\right|,$$ (26)

where $\left(x_{i},y_{i},z_{i}\right)$ denotes the coordinates of the i’th reconstructed point. The root mean square error of the radial deviations between the reconstructed points and the fitted spherical surface was also calculated as

$$\mathrm{RMSE}=\sqrt{\frac{1}{n}\overset{n}{\underset{i=1}{\sum }}E_{\text{radial},i}^{2}},$$ (27)

where n is the number of reconstructed points included in the analysis. The mean absolute error and standard deviation of the radial errors were also calculated. In addition, the fitted sphere diameter was compared with the known diameter of the reference target to assess dimensional agreement.

#### 3.1.3 Image acquisition procedure

Following geometric validation of the FPP system, the ocular specimen was positioned within the calibrated measurement area for image acquisition under controlled pressurization. Phase-shifted fringe patterns were then projected onto the ocular surface using the three-step, dual-frequency phase-shifting method described in Sec. 2.1. In this approach, three high-frequency and three low-frequency fringe patterns were sequentially projected unto the eyeball. The high-frequency fringe period was 18 projector pixels, whereas the low-frequency fringe period was 240 projector pixels. Although this projection sequence was ongoing, hydrostatic pressurization was initiated by manually adjusting the height of the fluid reservoir. This gradually increased the IOP and resulted in a corresponding expansion of the eyeball. This expansion and the resulting deformation of the projected fringe patterns were continuously recorded by the camera at a resolution of 960×720  pixels and a frame rate of ∼100  Hz. This provided dense temporal sampling of the changing surface despite possible variations in the manual height adjustment rate. To ensure that this continuous recording remained synchronized with the projected fringe patterns, the camera and projector were connected to an Arduino Uno through two separate digital output pins. The Arduino was programmed to generate synchronized timing signals for both devices. At the 100 Hz synchronization rate, each cycle lasted 10 ms and consisted of a digital HIGH signal of 1.25 ms followed by a digital LOW signal of 8.75 ms. This timing sequence was repeated continuously to coordinate the projection and image-acquisition events.

In total, the acquisition sequence yielded approximately 6000 raw fringe images. To convert these into 3D reconstructions, the images were first partitioned into discrete groups of six (three high frequency and three low frequency). Following this partitioning, the methodology described in Sec. 2.1 was applied to compute the wrapped phase maps for both the high and low-frequency components. Subsequently, the low-frequency phase was used to guide the unwrapping of the high-frequency phase, enabling recovery of the absolute phase distribution. This unwrapped phase was then used to generate the 3D reconstructions using the method described in Sec. 2.1, producing approximately 1000 surface reconstructions. Alongside these reconstructions, corresponding texture images were generated using the procedure described in Sec. 2.2, providing the intensity information required for subsequent displacement tracking.

#### 3.1.4 Displacement and strain computation

The texture images generated from FPP were used to compute in-plane displacement fields between deformation states. This was achieved by applying 2D DIC to the texture image sequence using the open-source Ncorr software package (version 1.2).^18^ In this process, the first texture image, corresponding to the undeformed configuration of the eye, was selected as the reference frame. All subsequent images were then compared with this reference to determine the displacement of surface features within successive frames. Using these displacement fields, the updated pixel locations on the displaced images were determined. These were then mapped onto the reconstructed 3D surfaces using the procedure described in Sec. 2.4, to obtain the corresponding spatial coordinates. With the spatial coordinates established for both the reference and deformed configurations, the 3D displacement fields (U,V,W) were computed using the procedure described in Sec. 2.4.

Following displacement recovery, the mechanical response of the ocular surface was characterized by computing the Green–Lagrange strain tensor, as formulated in Sec. 2.5. Given the approximately spherical geometry of the eye, the computed strain components were subsequently transformed from the Cartesian coordinate system to a spherical coordinate frame using the formulation described in Sec. 2.5.

The section that follows describes the results obtained from the outlined methodology.

## 4 Results

This section presents the results obtained using the proposed FPP–DIC measurement framework. It begins with a quantitative validation of the FPP system using a calibrated spherical target. The reconstructed 3D ocular surfaces during hydrostatic pressurization are then presented, followed by the corresponding displacement and strain measurements.

### 4.1 System-Level Validation of the FPP System

**Fig. 4:**
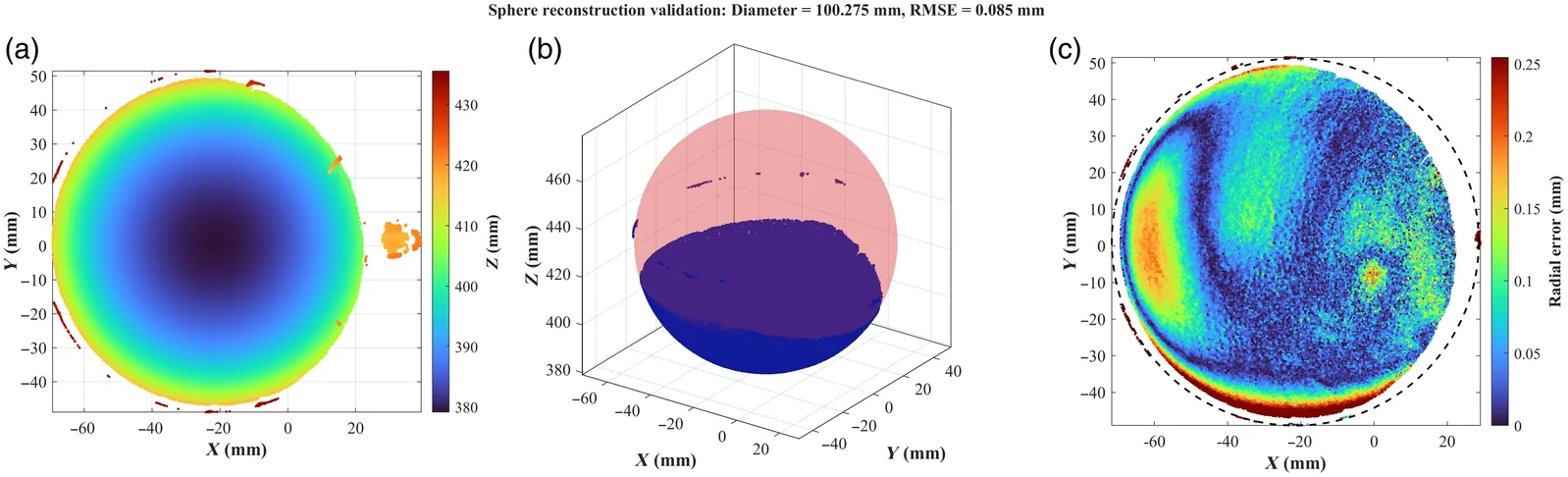
System-level geometric validation of the fringe projection profilometry (FPP) system using a calibrated spherical target with a known diameter of 100 mm. (a) Reconstructed depth map of the spherical target. (b) 3D reconstructed sphere (blue) overlaid with the RANSAC-fitted reference sphere (transparent red). (c) Spatial distribution of the radial error between the reconstructed points and the fitted spherical surface; the dashed circle indicates the projected boundary of the fitted sphere.

The results obtained from the FPP system geometric validation are presented in Fig. 4. As shown in Figs. 4(a) and 4(b), the reconstructed spherical surface closely followed the fitted reference geometry. The fitted sphere diameter was 100.2754 mm, corresponding to an absolute diameter difference of 0.2754 mm and a relative diameter error of 0.2754%.

The radial error distribution is shown in Fig. 4(c). The reconstructed points yielded a radial RMSE of 0.0848 mm, a mean absolute error of 0.0608 mm, and a standard deviation of 0.0591 mm relative to the fitted spherical surface. These results indicate sub-millimeter static geometric reconstruction error for the FPP system.

This quantitative validation supports the use of the FPP system for reconstructing the 3D ocular surface geometry required for the subsequent FPP–DIC displacement analysis.

### 4.2 3D Surface Reconstruction

**Fig. 5:**
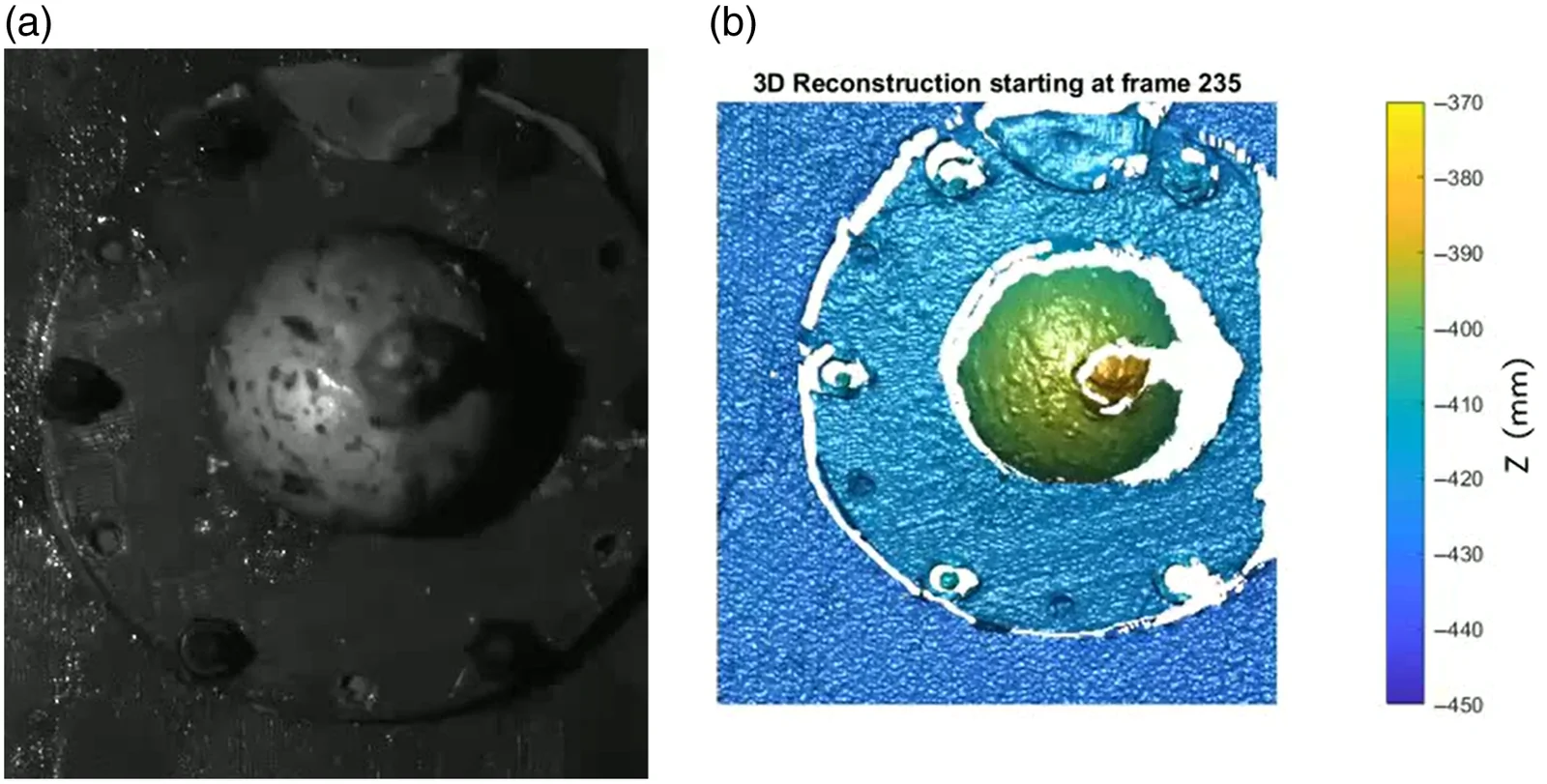
Representative still frame extracted from Video 1, showing (a) the grayscale texture image of the ocular surface and (b) the corresponding 3D reconstruction obtained using fringe projection profilometry (FPP). The reconstructed surface is visualized using a depth-based color map, where cooler blue tones indicate lower surface elevations and transitions toward green denote increasing height relative to the reference plane (Video 1, MP4, 321 KB [URI: https://doi.org/10.1117/1.JBO.31.8.086008.s1]).

Figure 5 presents a representative still frame extracted from Video 1, showing both the grayscale texture image and the corresponding 3D reconstruction of the ocular surface obtained using FPP. From this frame, it is qualitatively observed that the FPP system appears to recover the overall morphology of the ocular surface, with the continuous curvature of the scleral shell clearly preserved. The reconstructed surface exhibits no visible reconstruction artifacts, indicating that the reconstruction process maintains the underlying geometry without introducing distortions. Alongside that, surface features also remain well defined across the entire field of view, suggesting that both the global curvature and localized surface variations were captured with visually consistent detail.

To facilitate interpretation of the reconstructed geometry, a depth-based color map is applied to the surface. In this representation, cooler blue tones correspond to lower surface elevations relative to the reference plane, whereas transitions toward lighter blue and green indicate increasing surface height. This mapping ensures that variations in-surface elevation are readily distinguishable. As such, the spatial distribution of the reconstructed geometry can be clearly visualized within just a single frame.

To verify that depth changes observed in Video 1 were primarily associated with hydrostatic pressurization of the ocular specimen rather than motion of the mounting system, a stationary planar region on the mount was analyzed while excluding the eyeball. A reference plane was fitted to the selected region in the first image frame, and the height differences of the corresponding reconstructed points were evaluated across the subsequent frames. The results showcased that the mean height change remained close to zero, ranging from −0.0071 to 0.0146 mm. This indicates that the mounting surface did not undergo a systematic frame-to-frame shift during image acquisition. Although local height differences were present within the stationary region, the RMS height difference ranged from 0.2423 to 0.5943 mm.

**Fig. 6:**
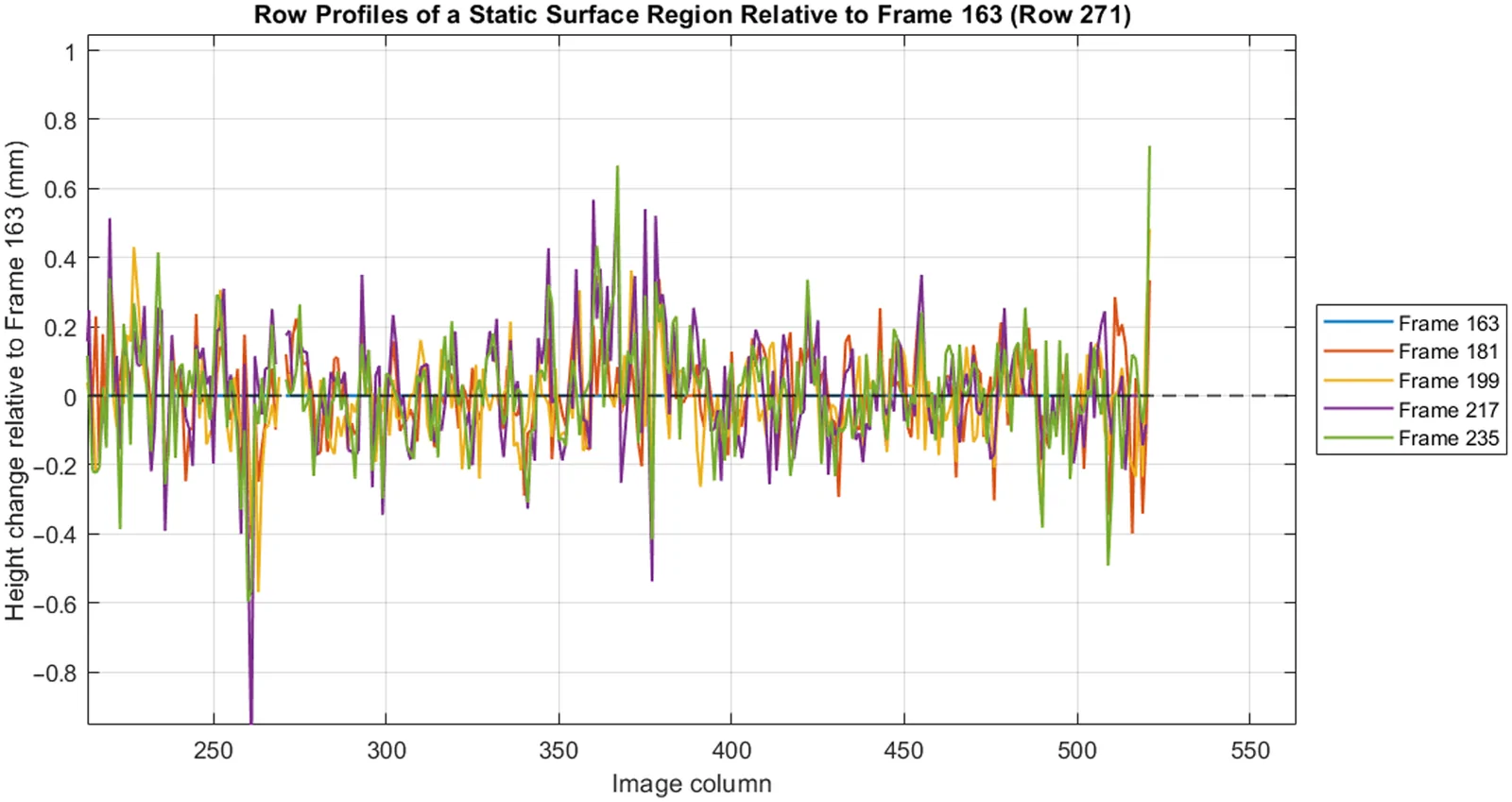
Representative height profiles along a selected row of a stationary mounting-surface region in Video 1. The profiles show the reconstructed height change for representative frames relative to Frame 163, which was used as the reference frame. Although local fluctuations are present, the profiles remain centered around zero, indicating spatially localized reconstruction variation rather than a systematic displacement of the mounting system.

To further examine these local variations, height values along the same horizontal line in the stationary region were compared across representative frames, as shown in Fig. 6. The profiles fluctuated locally above and below the zero-height-change line. However, they did not show a consistent upward or downward shift across frames. These results indicate that the background variations were localized rather than caused by motion of the mounting system. Therefore, the progressive surface changes observed in Video 1 are attributed primarily to hydrostatic pressurization-induced deformation of the eyeball.

Overall, these results indicate that the FPP system has the potential to accurately reconstruct the ocular surface and resolving its surface height variations. They also indicate that the progressive height changes observed in Video 1 were primarily caused by hydrostatic pressurization of the ocular specimen rather than motion of the mounting system.

### 4.3 2D Displacement Fields

**Fig. 7:**
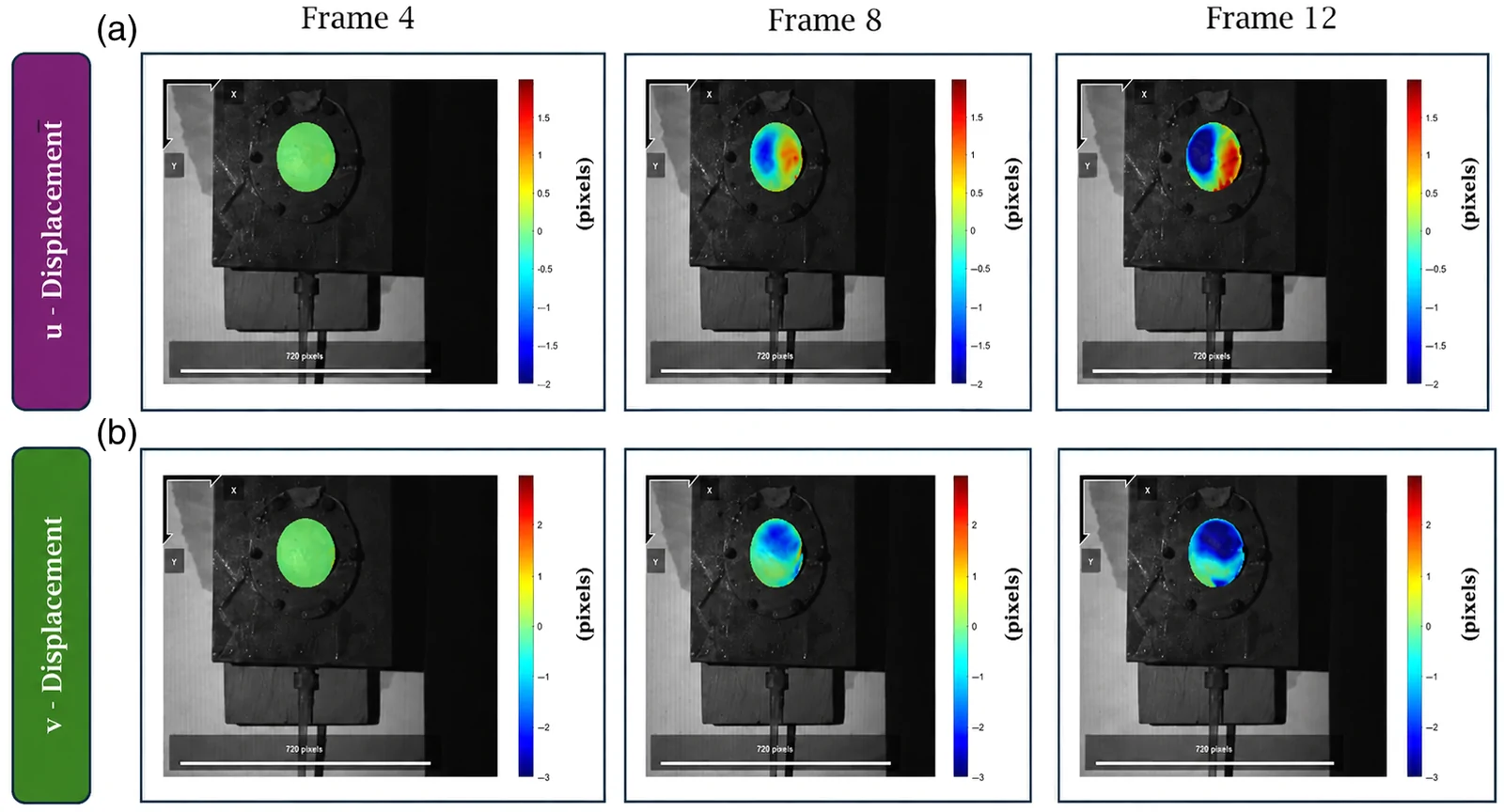
Representative in-plane displacement fields obtained from digital image correlation (DIC) at three stages of ocular deformation. The columns correspond to selected frames during pressurization (frame 4, frame 8, and frame 12), illustrating the temporal evolution of deformation. Panel (a) shows the horizontal displacement component (u), whereas panel (b) shows the vertical displacement component (v). The colored overlays represent the measured displacement magnitude within the region of interest, mapped onto the grayscale texture image of the eyeball. The color bars indicate displacement magnitude for each frame. The results illustrate the progressive increase and spatial redistribution of surface displacement as internal pressure increases.

Following 3D reconstruction 2D DIC was performed on the texture images. The in-plane displacement maps obtained from this DIC analysis are shown in Fig. 7. Theses maps illustrate the evolution of the horizontal (u) and vertical (v) displacement components over successive frames. The color scale represents the magnitude and direction of displacement within the image plane, with warmer colors indicating positive displacement and cooler colors indicating negative displacement.

At the initial state, the displacement fields exhibit negligible variation, consistent with the undeformed reference configuration. However, as pressurization progressed, displacement patterns became increasingly pronounced across the ocular surface. The displacement magnitude increased progressively with intraocular pressure, indicating a continuous expansion of the surface. Overall, these patterns suggest a pressure-dependent deformation trend, with both u and v components evolving consistently over time.

### 4.4 3D Displacement Fields

**Fig. 8:**
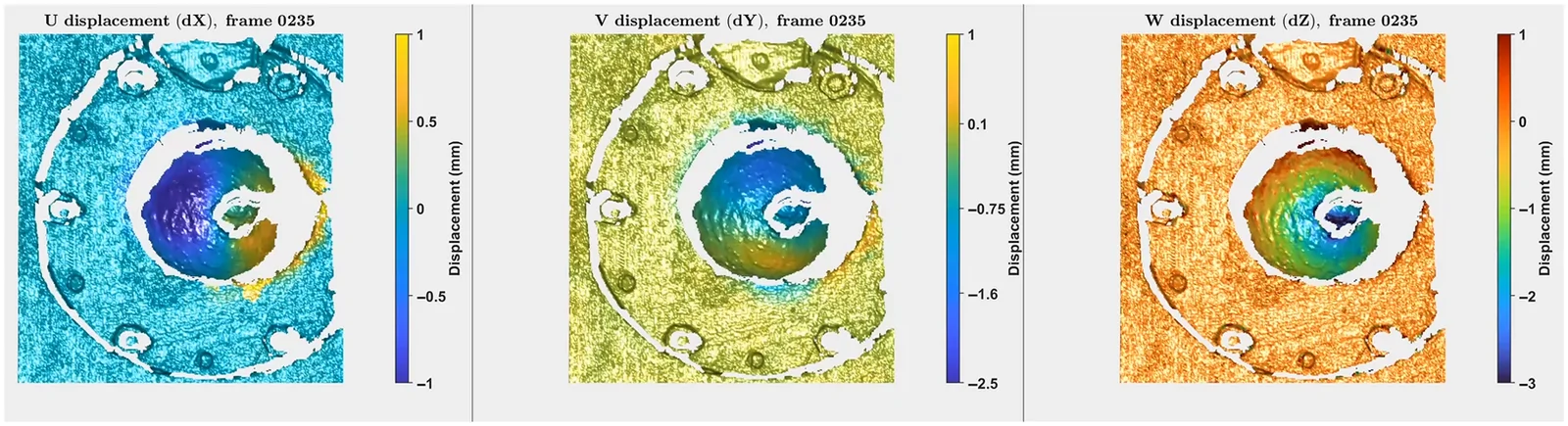
Representative still frame extracted from Video 2, showing the full-field displacement of the ocular surface mapped onto the reconstructed 3D geometry. The in-plane displacement fields obtained from 2D DIC are integrated with the fringe projection–based reconstruction to recover the 3D displacement components, namely, U, V, and W, corresponding to motion along the x, y, and out-of-plane direction z, respectively. Each displacement component is visualized using a color map, where variations in color indicate both the magnitude and direction of surface motion relative to the reference configuration. The U and V fields describe lateral surface motion, whereas the W field captures deformation normal to the ocular surface (Video 2, MP4, 4.32 MB [URI: https://doi.org/10.1117/1.JBO.31.8.086008.s2]).

Figure 8 presents a representative still frame from Video 2. It illustrates the 3D displacement components (U,V,W) of the ocular surface obtained through the fusion of FPP and DIC at a selected stage of pressurization. To facilitate the interpretation of this static visualization, color maps were applied to each component to represent both the magnitude and direction of surface motion. In these maps, warmer colors denote displacement in the positive direction, whereas cooler colors indicate displacement in the negative direction. Furthermore, because each component exhibits a distinct range of values, their respective color scales are defined independently. This approach ensures that the spatial variations within each individual displacement field are more clearly resolved.

Applying this visualization reveals clear spatial variation across the ocular surface at the selected frame. Specifically, the W component exhibits pronounced out-of-plane deformation, whereas the U and V components capture the lateral motion along the surface. Notably, the regions of maximum displacement differ between these components, indicating that the deformation is not uniform. Instead, distinct areas of the surface experience varying levels of displacement, a phenomenon effectively highlighted by the independent color mapping.

### 4.5 Strain Distribution

**Fig. 9:**
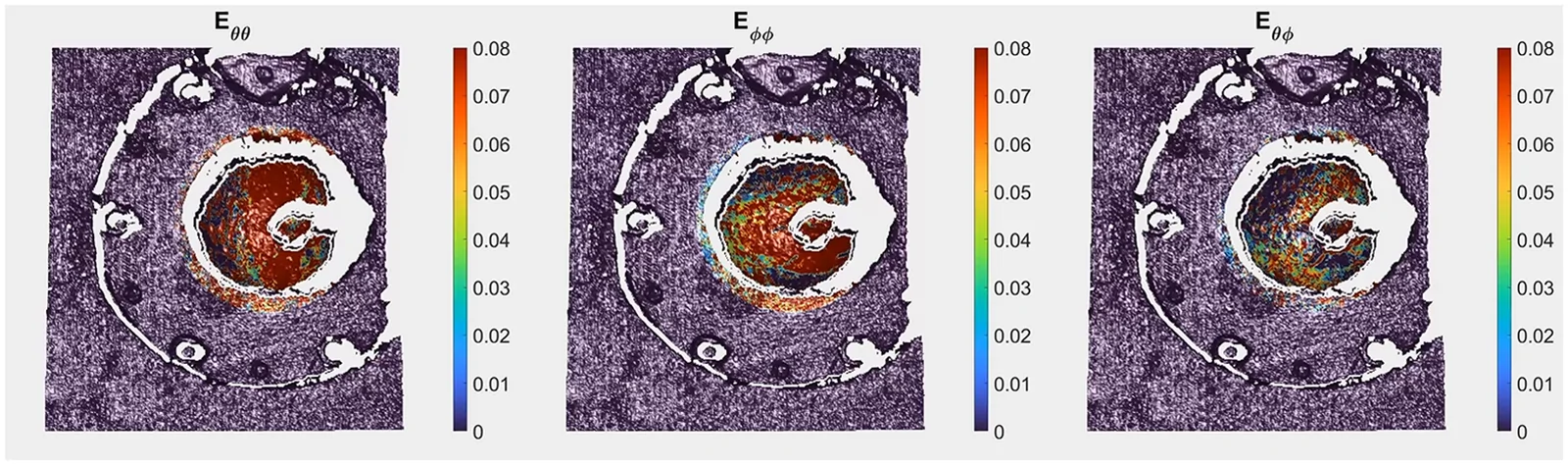
Representative still frame extracted from Video 3, showing the spatial distribution of strain components mapped onto the reconstructed 3D ocular surface. The strain fields are computed from the recovered displacement vectors using the Green–Lagrangian formulation and subsequently expressed in the spherical coordinate frame. The components $E_{\theta \theta }$, $E_{\theta \phi }$, and $E_{\phi \phi }$ correspond to circumferential strain, in-plane shear strain, and meridional strain along the curved ocular surface, respectively. Each component is visualized using a color map, where variations in color indicate the magnitude and sign of strain relative to the reference configuration (Video 3, MP4, 6.17 MB [URI: https://doi.org/10.1117/1.JBO.31.8.086008.s3]).

Following the computation of the 3D displacement field, the corresponding strain distribution across the ocular surface was evaluated. The results of this evaluation are depicted in Fig. 9. This figure presents a representative still frame extracted from Video 3, illustrating the spherical strain components $E_{\theta \theta }$, $E_{\theta \phi }$, and $E_{\phi \phi }$ at a selected stage of pressurization. To facilitate interpretation, color maps are applied to each strain component to represent the magnitude of strain. In these maps, warmer colors indicate higher strain magnitudes, whereas cooler colors correspond to lower strain magnitudes.

## 5 Discussion

This study set out to assess whether an FPP–2D DIC framework can serve as a viable noncontact approach for ocular surface analysis. Specifically, it examines the framework’s ability to capture 3D surface geometry and recover 3D displacement of the ocular surface under controlled pressurization. The results indicate that the proposed modality has the potential to capture the evolving surface geometry and associated displacement and strain fields. Thus, it suggests that the framework can serve as a promising tool for capturing the mechanical response of the ocular surface. The potential of the framework is first reflected in the observed performance of the FPP system in reconstructing the ocular surface geometry. However, before applying the framework to the ocular specimen, the geometric accuracy of the FPP system was quantitatively evaluated using a calibrated spherical target. The validation showed sub-millimeter reconstruction accuracy, providing confidence that the system could reliably capture the complex geometry of the ocular surface. Building on this validation, the framework was then applied to reconstruct the ocular surface during hydrostatic pressurization. The results show that the reconstructed surfaces preserved the overall morphology of the eye without visible reconstruction artifacts, indicating that the system was able to resolve the underlying geometry. Importantly, this reconstruction quality is maintained under pressurized conditions, where the ocular surface undergoes dynamic deformation. This is evidenced by the ability of the FPP system to consistently produce stable and continuous surface representations throughout the deformation process, despite the continuous change in ocular geometry during image acquisition. This behavior is particularly relevant in comparison with conventional imaging modalities, for which motion during sequential acquisition can introduce reconstruction errors or reduced fidelity, thereby affecting measurement reliability.^23^

A second indication of the framework’s potential is reflected in its ability to recover displacement and strain fields from the reconstructed geometry. These fields provide direct insight into how the ocular surface responds mechanically to pressurization, enabling assessment of deformation behavior beyond geometric reconstruction alone. That being said, it becomes essential to evaluate whether the recovered displacement and strain patterns are physically meaningful. Although no quantitative validation was performed, as this study was designed as a feasibility investigation, a qualitative evaluation of the obtained results was conducted. This evaluation revealed three key observations that support the physical and biological relevance of the recovered displacement and strain fields, particularly when interpreted in the context of established literature.

First, the displacement fields exhibit clear spatial heterogeneity across all components (U, V, W), as shown in Fig. 8. This nonuniform distribution indicates that deformation varies across different regions of the ocular surface rather than occurring uniformly. Such behavior is well supported in the literature,^24^^,^^25^ where the eye is recognized as a structurally heterogeneous organ composed of regions with varying thickness, curvature, and material properties, particularly within the sclera and surrounding tissues. These structural variations lead to region-dependent mechanical responses under pressure.^24^^,^^25^ The ability of the proposed framework to capture this heterogeneous deformation pattern therefore suggests that it is resolving physically meaningful behavior consistent with known ocular biomechanics.

Second, the strain fields reveal a localized concentration of maximum strain near the optic nerve head (ONH). This observation is also consistent with established biomechanical studies, which identify the ONH as a mechanically sensitive region due to the transition between the relatively stiff scleral tissue and the more compliant neural tissues of the optic nerve.^1^ This structural discontinuity leads to stress and strain concentration under intraocular pressure loading. The identification of this localized strain peak in the present results further supports the capability of the framework to capture biomechanically relevant deformation features.

Third, the spatial distribution of the strain components exhibits distinct directional characteristics. The circumferential strain ($E_{\theta \theta }$) forms a ring-like pattern aligned with the curvature of the ocular surface, whereas the meridional strain ($E_{\phi \phi }$) is more broadly distributed. This behavior aligns with classical descriptions of deformation in pressurized curved structures, where circumferential stretching dominates along the surface perimeter and meridional deformation propagates across the structure. Similar patterns have been reported in prior studies of ocular mechanics, where curvature-driven loading results in anisotropic strain distributions.^26^ The agreement between these expected deformation modes and the observed results provides further qualitative evidence of the physical validity of the framework.

Taken together, these findings indicate that the proposed FPP–DIC framework has the potential to capture full-field 3D deformation of the ocular surface with sufficient spatial resolution and consistency. More importantly, the results indicate that the measured displacement and strain fields reflect realistic biomechanical behavior, supporting the feasibility of this approach as a noncontact tool for ocular surface deformation analysis.

## 6 Conclusion

This study indicates the feasibility of a noncontact optical framework that integrates FPP and 2D DIC to capture full-field 3D deformation of an ocular surface under controlled pressurization. In addition, it provides qualitative insight into the associated displacement and strain patterns. These insights indicate that the proposed approach can recover continuous surface geometry and spatially varying displacement fields in a manner consistent with expected biomechanical behavior.

Beyond demonstrating feasibility, these results highlight the potential of the framework as an alternative to conventional imaging modalities. In particular, its ability to capture dense, full-field deformation under dynamic conditions, combined with its relatively low system complexity and cost, suggests that it may offer a practical and scalable solution for ocular surface analysis.

Despite the potential of the proposed framework, several limitations should be acknowledged. First, the present study was conducted using a limited dataset consisting of a single porcine eyeball. This restricts the generalization of the findings and prevents assessment of biological variability across samples. In addition, the experiments were performed on an *ex vivo* specimen under controlled pressurization conditions. Although this setting enabled evaluation of the proposed measurement framework, it does not fully reproduce the physiological conditions encountered *in vivo*, where natural ocular motion, tear-film behavior, and tissue variability may introduce additional measurement challenges.

Beyond these study-level limitations, the current imaging configuration also imposes practical constraints. The single-view camera–projector arrangement limited measurement to the portion of the ocular surface that was simultaneously visible to the camera and adequately illuminated by the projector. In particular, in Fig. 5, a locally unreconstructed region was observed near the optic nerve head. Because the optic nerve head protrudes from the posterior ocular surface, it partially blocked the projected fringes from reaching the surface region directly behind it relative to the projector direction. As a result, that region received insufficient fringe information for reliable phase recovery and 3D reconstruction. This limitation could be reduced in future work by introducing additional projector views to improve surface coverage and reduce occlusion-related data loss.

In addition to the surface-coverage limitation, the current optical measurement system is also sensitive to the local reflectance properties of the ocular surface. Because the ocular surface is curved and moist, localized variations in reflectance and specular reflections may occur under certain imaging conditions. These effects can reduce fringe contrast or locally saturate the captured images, thereby reducing the reliability of phase recovery in affected regions. Although sufficient fringe visibility was obtained in the present measurements, future implementations could incorporate reflection-management strategies, including polarization-based glare suppression, adaptive exposure control, and fringe-quality masking.

Finally, although visible motion blur was not observed under the gradual pressurization rate and approximately 100 Hz acquisition rate used in this study, the sequential nature of phase-shifting measurements may become more susceptible to motion-related phase errors when the ocular surface deforms more rapidly. To address this in future work, faster synchronized acquisition, higher-speed phase-measurement strategies, and controlled dynamic validation experiments could be adopted to extend the framework to more demanding and physiologically relevant measurement conditions.

## Supplementary Material

10.1117/1.JBO.31.8.086008.s1

10.1117/1.JBO.31.8.086008.s2

10.1117/1.JBO.31.8.086008.s3

## Data Availability

The datasets generated and/or analyzed during the current research alongside the code used for analysis are available at Google Drive: https://drive.google.com/file/d/1aC9nX_CkpNcae-5VLFA-UIJk8FNAMUZ2/view
